# Viral respiratory tract infections in young children with cystic fibrosis: a prospective full-year seasonal study

**DOI:** 10.1186/s12985-019-1208-7

**Published:** 2019-09-03

**Authors:** Mathilde Eymery, Florence Morfin, Anne Doleans-Jordheim, Marie Perceval, Camille Ohlmann, Catherine Mainguy, Philippe Reix

**Affiliations:** 1 Service de pneumologie pédiatrique et CRCM enfant, Hôpital Femme Mère Enfant, Hospices civils de Lyon, Bron, France; 2 Laboratory of Virology, Institut des Agents Infectieux, Groupement Hospitalier Nord, F69317 Lyon, France; 3 Centre National de Référence des virus respiratoires France Sud, Hospices Civils de Lyon, Lyon, France; 4 Faculté de Pharmacie, CIRI, Inserm U1111 CNRS UMR5308, Virpath, Univ Lyon, Université Lyon 1, Lyon, France; 5 Equipe de Recherche, Bactéries Pathogènes Opportunistes et Environnement, UMR CNRS 5557 Ecologie Microbienne, Université Lyon 1 & VetAgro Sup, Villeurbanne, France; 6 Laboratory of Bacteriology, Institut des Agents Infectieux, Groupement Hospitalier Nord, Hospices Civils de Lyon, F69317 Lyon, France; 7 UMR 5558 (EMET). CNRS, LBBE, Université de Lyon, Villeurbanne, France; 8 Centre de ressources et de compétence pour la Mucoviscidose, 59 boulevard Pinel, 69677 BRON CEDEX, France

**Keywords:** Children, Respiratory virus, Cystic fibrosis

## Abstract

**Background:**

Viral respiratory tract infections are common during early childhood. How they impact cystic fibrosis lung disease history in young children is poorly known. The principal aim of our study was to determinate respiratory tract infections frequency in this cystic fibrosis young population. Secondary outcomes were nature of viral agents recovered and impact of such infections.

**Methods:**

We conducted a prospective cohort study of 25 children affected by cystic fibrosis and aged less than 2 years. Nasal samplings were taken systematically monthly or bimonthly with additional samples taken during respiratory tract infections episodes. Ten pathogens were tested by a combination of five duplex RT-PCRs or PCRs: *influenza A* and *B*, *respiratory syncytial virus* (RSV), *metapneumovirus* (MPV), *rhinovirus/enterovirus* (RV/EV)), *coronavirus* (HKU1, NL63, 229E and OC43), *parainfluenza virus* (1–4), *adenovirus* and *bocavirus* (Respiratory Multi-Well System MWS r-gene®, BioMérieux, Marcy l’Étoile, France). Cycle thresholds (CTs) were reported for all positive samples and considered positive for values below 40. Quantitative variables were compared using a nonparametric statistical test (Wilcoxon signed rank for paired comparisons). Pearson’s correlation coefficient (r) was used to assess relationships between two variables. Statistical analyses were performed using SAS v9.4 (SAS Institute, Cary, NC, USA) or GraphPad Prism V6.00 (GraphPad Software, La Jolla, CA, USA). The significance level was set at 0.05.

**Results:**

The mean age at inclusion was 9.6 ± 6.7 months. The patients had 3.4 ± 1.7 respiratory tract infections episodes per child per year. Forty-four respiratory tract infections (69%) were associated with virus: *rhinovirus* and *enterovirus (RV/EV)* were implied in 61% of them and *respiratory syncytial virus* (RSV) in 14%. Only one patient required hospitalization for lower respiratory tract infections. 86% of the patients were treated by antibiotics for a mean of 13.8 ± 6.2 days. RSV infections (*n* = 6) were usually of mild severity.

**Conclusions:**

Respiratory tract infections in young children with cystic fibrosis were of mild severity, rarely requiring hospitalization. Unsurprisingly, *RV/EV* were the most frequent agents. RSV-related morbidity seems low in this population. This raises the question of the usefulness of RSV preventive medication in this young population.

## Background

Cystic fibrosis (CF) is a rare genetic condition affecting more than 90,000 people worldwide. Despite implementation of neonatal newborn screening programs in some countries and improvement in patient care over the last few years, CF-related lung disease remains the main cause of morbidity-mortality in these patients. The role of bacterial pathogens such as *Staphylococcus aureus* (SA) and *Pseudomonas aeruginosa* (PA) has been extensively studied for several decades whereas the respiratory viruses pathogenesis remained little explored. The availability of new diagnostic molecular tests to detect virus infections has recently boosted the interest in evaluating their impact during pathological conditions such as CF in children [1–9] and in adults [10–15].

There is however a critical lack of data describing viral respiratory tract infections (RTIs) in young children with CF aged less than 2 years, when RTIs are particularly frequent and potentially more severe at this early period of life. Some studies conducted in the late 1980s and 1990s have drawn a pessimistic scenario of viral RTIs in this vulnerable population that is no longer observed. In these seminal studies, clinical manifestations were described as severe, frequently requiring hospitalization and prolonged oxygen supply or mechanical ventilation [16–18]. Some patients were described as acquiring pathogens such as PA in the weeks or months following RTI [18, 19]. At that time, *respiratory syncytial virus* (RSV) was found to be the most frequently recovered virus, but diagnostic tools were limited to immunological assays and viral culture, thus limiting the spectrum of virus that may be recovered. Recent studies have shown that RSV epidemics may be associated with the occurrence of more pulmonary exacerbations [20], but RSV is rarely responsible for hospitalization because of respiratory events in CF patients [21, 22].

We designed a prospective cohort study to better explore the frequency of RTI, along with their severity, the nature of viral agents recovered and the associated short-term outcomes.

## Material and methods

### Patient recruitment

Between March 2015 and April 2016, 33 families were contacted. Eight refused to participate (three because repeated nasal swab samplings were required, two because of the overall burden of the study, two for unexplained reasons, one for a language barrier issue). Finally, 25 young children with CF aged less than 24 months were recruited to participate in the study. All had confirmed CF based on compatible clinical features associated with two positive sweat tests (sweat chloride concentration ≥ 60 mmol/L) and/or two CFTR gene mutations. None received palivizumab because it is not used in our current practice.

### Study design, ethics, objectives and outcome measures

This was a prospective cohort study conducted in a single pediatric center (Lyon, France). The study was approved by the local Institutional Review Board (Comité Consultatif de Protection des Personnes dans la Recherche Biomédicale, Lyon; No. 2014-AO1387640). Parents or legal guardians gave their assent and signed an informed consent form before their children entered the study.

The primary outcome measure was the frequency of RTIs per year. The secondary outcomes measured during the year of follow-up were: (1) virus identification by multiplex PCR; (2) number of hospitalizations, number of oral/intravenous antibiotic courses, steroids, bronchodilator use, number of days of parental work absenteeism, unscheduled outpatient visits; (3) difference in weight Z-scores between the start and the end of the study; (4) comparison between virus-negative and -positive RTIs for age and weight Z-score at inclusion, difference in weight Z-scores between the beginning and end of the study period, total number of RTIs, cumulative number of days of antibiotics; (5) percentage of samples positive for PA and SA out of the the total number of samples taken routinely during the study, new isolation of a pathogen.

### RTI identification, nasal swab sampling and viral analysis

It is routine practice in France for chest physiotherapists to visit patients at home to ensure regular chest physiotherapy and respiratory surveillance. As already reported [23] we thought that proper nasal sampling and filling out diaries by parents may be difficult to perform repeatedly over a long period of time; chest physiotherapists were therefore involved in the study to take nasal samples and recognize RTIs. Parents and chest physiotherapists were instructed to recognize RTI using a modified clinical score already used in a previous study conducted by van Ewijk and al in young children with CF [9]. There, the score was systematically performed twice weekly by parents, while in our study it was used on request by parents and physiotherapists to detect or confirm a possible RTI episodes revealed by the occurrence of symptoms. Briefly, Upper and Lower respiratory tract infection (URTI and LRTI) symptoms as well as general signs such as fever were recorded and a score calculated. In case of doubt, parents could ask the research nurse (by phone) or their chest physiotherapist (during the home visit) to confirm their findings. When the score was over 2, a nasal sample was collected at home by the chest physiotherapist within a maximum 3 days after the beginning of the symptoms, or at the center when the episode was coinciding with a visit.

Furthermore, nasal swabs were systematically scheduled (even in absence of RTI signs) at the following frequencies depending on viral seasonality: every month from September 1st to March 31st and every 2 months from April 1st to October 31st during the year following inclusion in the study.

Nasopharyngeal samples were taken according to a recently described procedure using swabs (Sigma-Virocult® M40 compliant. MWE. Corsham Wiltshire, UK) after saline instillation [24].

Respiratory specimen sampling (pharyngeal swabs) for bacterial cultures was performed at each center visit (four to six samples per patient per year depending on the age at inclusion). Bacterial cultures were performed on specific medias in order to identify major CF pathogens such as SA or PA. Colonies morphology, gram staining were than used for a first identification of each bacterial species. Than a MALDI-TOF-MS approach was done for the final identification.

Ten pathogens were tested by a combination of five duplex RT-PCRs or PCRs: *influenza A* and *B*, *respiratory syncytial virus* (RSV), *metapneumovirus* (MPV), *rhinovirus/enterovirus* (RV/EV)), *coronavirus* (HKU1, NL63, 229E and OC43), *parainfluenza virus* (1–4), *adenovirus* and *bocavirus* (Respiratory Multi-Well System MWS r-gene®, BioMérieux, Marcy l’Étoile, France). Cycle thresholds (CTs) were reported for all positive samples and considered positive for values below 40.

### Statistical analysis

Quantitative variables are reported as mean ± standard deviation. Quantitative variables were compared using a nonparametric statistical test (Wilcoxon signed rank for paired comparisons). Pearson’s correlation coefficient (r) was used to assess relationships between two variables. Statistical analyses were performed using SAS v9.4 (SAS Institute, Cary, NC, USA) or GraphPad Prism V6.00 (GraphPad Software, La Jolla, CA, USA). The significance level was set at 0.05.

## Results

### Patient characteristics at inclusion

Twenty-five young children with a mean age of 9.6 ± 6.7 months (range, 2–22.9 months) were included (Table 1). Half of them had siblings in their immediate family environment. Half attended exclusively family daycare. None received palivizumab and 22 patients (88%) were vaccinated against influenza virus. Sixty percent had commensal flora at inclusion.

**Table 1:** Patient characteristics at inclusion

Number of patients	25
Gender (male/female)	15/10
Premature birth^a^ *n* (%)	5 (20%)
Season at birth^b^	
Spring	6 (24%)
Summer	6 (24%)
Fall	5 (20%)
Winter	8 (32%)
Maternal smoking during pregnancy	4 (16%)
Maternal atopy	6 (24%)
Parental smoking	15 (60%)
Siblings	
0	12 (48%)
1	8 (32%)
≥2	5 (20%)
Daycare attendance^c^	Family only: 13 (52%)
	Small group: 8 (32%)
	Collective: 4 (16%)
Demographics at inclusion	
*CFTR* genotype with	
No CFTR function	21 (84%)
Residual CFTR function	5 (16%)
Unknown consequences	0 (0%)
Age at inclusion (months)	9.4 ± 6.7
Range	2–22.9
Anthropometrics (Z-scores)	
Weight	−1.3 ± 1.4
Height	−1.1 ± 1.5
Vaccination coverage rates	
DTPa-IPV/Hib^d^	25 (100%)
Influenza	22 (88%)
RSV prophylaxis (palivizumab)	0 (0%)
Bacteria recovered at inclusion	
Oropharyngeal flora	15 (60%)
*Staphylococcus aureus*	8 (32%)
*Pseudomonas aeruginosa*	1 (4%)
*Haemophilus influenzae*	1 (4%)
Virus recovered at inclusion	
None	10 (40%)
*RV/EV*	7 (28%)
*Bocavirus*	4 (16%)
*Influenzae*	2 (8%)
*Metapneumovirus*	1 (4%)
*Cytomegalovirus*^e^	1 (4%)
*RSV*	0 (0%)

Results are presented as mean ± SD or *n* (%)

^a^Birth before 37 weeks of gestational age

^b^Winter: 22/12 to 19/03, spring: 20/03 to 20/06, summer: 21/06 to 21/09; fall: 22/09 to 21/12

^c^Small group means fewer than four children, collective daycare means more than or equal to four children

^d^DTPa- IPV/Hib vaccine: diphtheria-tetanus, acellular pertussis, inactivated poliomyelitis, adsorbed conjugated *Haemophilus influenzae* type b vaccine

^e^Cytomegalovirus was not part of the viral panel. It was identified from a respiratory sample by viral culture

### Primary outcome: RTI frequency

**Fig. 1:**
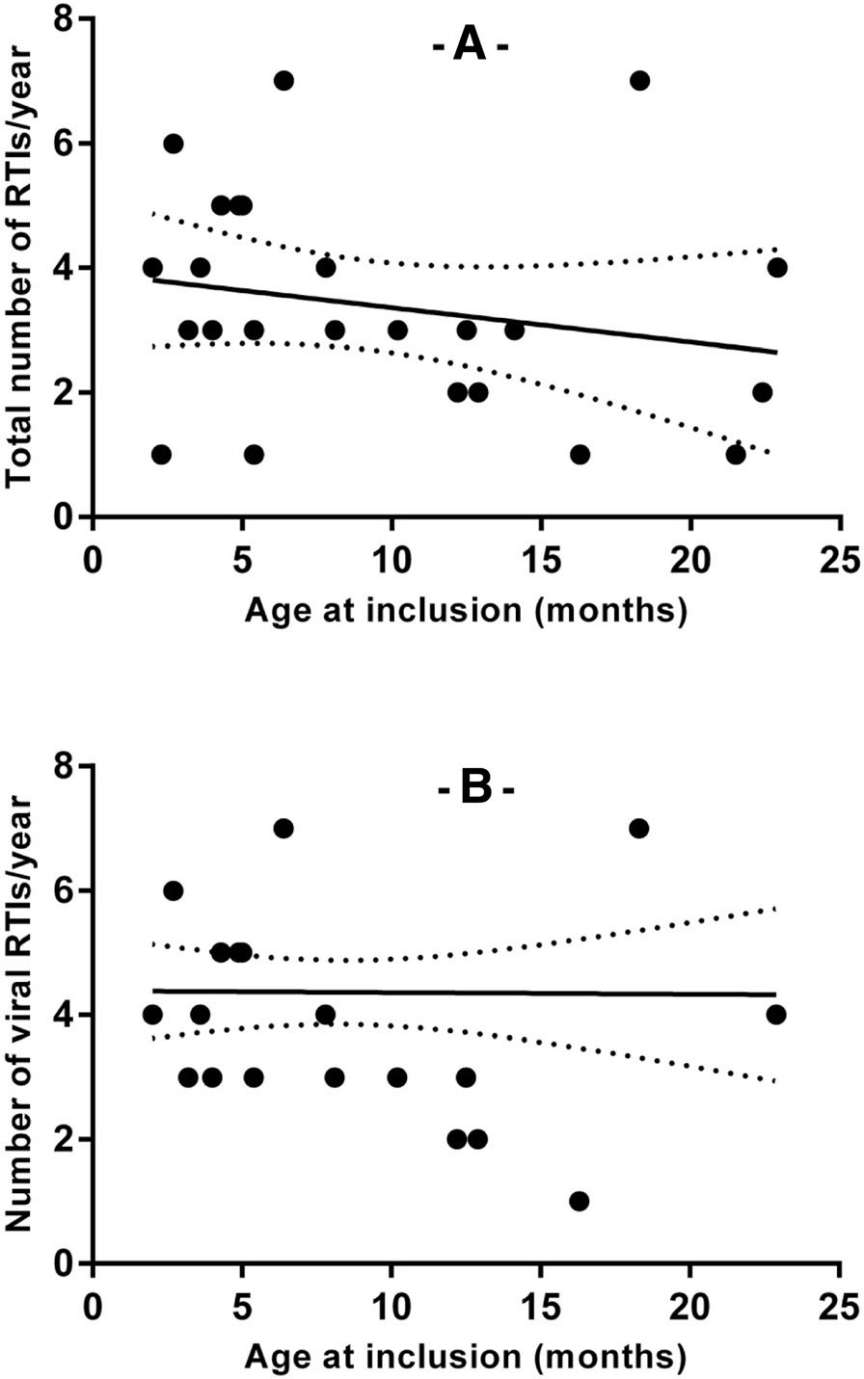
**a** Correlation between age at inclusion and total respiratory tract infection (RTI) episodes; **b** Correlation between age at inclusion and virus-positive respiratory tract infection (RTI) episodes

Eighty-five RTIs were identified for an average of 3.4 ± 1.7 RTIs per child per year. Virally proven RTIs were identified at an average of 1.8 ± 1.5 per child per year. There was no correlation between age at inclusion and the total number of RTIs (Pearson correlation, *r* = − 0.24, *p* = 0.2353) nor virus-positive RTIs (Pearson correlation, *r* = 0.065, *p* = 0.6710) (Fig. 1 correlation between age at inclusion and a. total respiratory tract infections or b. virus positive respiratory tract infection episodes). There were no specific clinical features related to viral-positive RTIs.

### Secondary outcomes

#### Viruses recovered during RTI

**Fig. 2:**
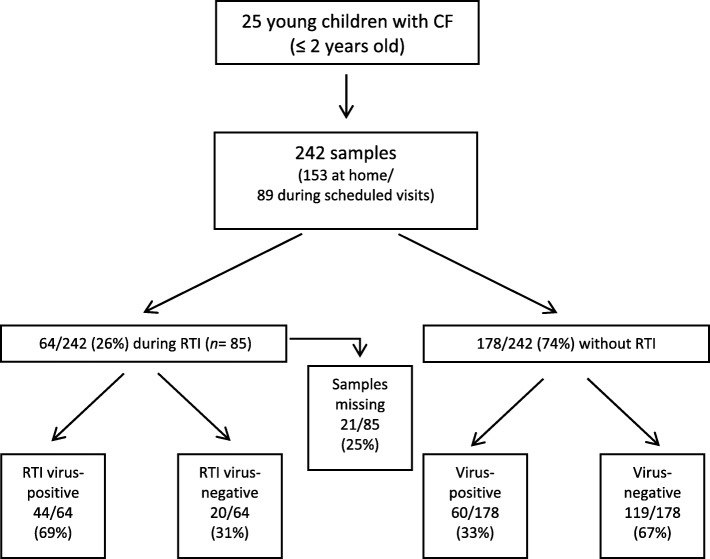
Flow chart of virus sample results

During the year of follow-up, 242 samples were collected (153 at home and 89 during a visit to the center), a mean of 9.7 ± 2.1 samples per patient. Out of the 85 RTIs clinically identified, 64 samples (75%) were collected during symptoms occurrence, while 21 samples were not realized. Out of 64 RTIs sampled, 44 (69%) were associated with the identification of a viral agent (Fig. 2 flow chart of virus sample results).

The viruses recovered during viral RTIs were *RV/EV* (27/44; 61%), *RSV* (6/44; 14%), *adenovirus* (6/44; 14%), *influenza* (4/44; 9%), *bocavirus* (3/44; 7%), *parainfluenzae* (2/44; 5%) and *metapneumovirus* (1/44; 2%). Eight viral co-infections were identified during RTIs; one of them required hospitalization (Table 2).

**Table 2:** Viruses recovered during RTI and without RTI

	During RTI (*n* = 44)	Without RTI (*n* = 60)
RV/EV	27 (61%)	31 (52%)
Adenovirus	6 (14%)	9 (15%)
RSV A/B	6 (14%)	2 (3%)
Influenza A/B	4 (9%)	1 (2%)
Bocavirus	3 (7%)	13 (22%)
Parainfluenza 1–4	2 (5%)	–
Metapneumovirus	1 (2%)	1 (2%)
Coronavirus	–	9 (15%)
Co-infections^a^	8 (20%)	8 (13%)

^a^co-infections during RTI: rhinovirus associated with adenovirus (*n* = 2), RSV (*n* = 1), bocavirus (*n* = 1); bocavirus associated with influenza (*n* = 1), adenovirus (*n* = 1) and adenovirus associated with RSV (*n* = 3)

Co-infections without RTI: rhinovirus associated with adenovirus (*n* = 2), bocavirus (*n* = 2), coronavirus (*n* = 1); bocavirus associated with adenovirus (*n* = 1), influenza (*n* = 1), cytomegalovirus (*n* = 1)

For the six patients presenting a RSV infection, the clinical progression was uncomplicated (Table 3).

**Table 3:** RSV infected patient’s characteristics

Patient number	Age at inclusion (months)	Age at RSV infection (months)	Requirements for extra-care			
			Hospitali-zation/ Oxygen supply	Antibiotics	SABD^c^	Oral steroids
1	16.3	22.9	No/No	Yes	Yes	No
2	8.1	19.6	No/No	Yes	Yes	Yes
3	4.0	12.8	No/No	Yes	Yes	Yes
4	2.0	14.1^a^	No/No	Yes	No	No
5	2.7	4.5^b^	No/No	Yes	No	No
6	3.2	12.3^a^	No/No	Yes	No	Yes

^a^co-infected with RVh

^b^co-infected with adenovirus

^c^*SABD* short acting bronchodilators

#### Viruses recovered without RTI

One-third of the viral samples collected outside RTI episodes were virus-positive (Table 2). Compared to those recovered during RTI, the proportions were quite similar for RV, adenoviruses and metapneumovirus. However, a smaller proportion of RSV and influenza but a higher proportion of bocavirus and coronavirus were found in nasal swabs collected outside RTI periods.

#### Comparison of virus-positive and -negative RTI outcomes (Table 4)

No differences were found between virus-positive and -negative RTIs among the following outcome measures: age at inclusion, weight Z-score at inclusion, difference of weight Z-scores between the beginning and end of the study period, total number of RTIs and cumulative number of days of antibiotics.

**Table 4:** Comparison of outcomes in virus-positive and -negative RTIs

Outcome measures	Virus (−) RTI	Virus (+) RTI	*P*-value
Number of episodes	(*n* = 20)	(*n* = 44)	
Age at inclusion (months)	7.1 ± 1.4	8.3 ± 0.9	0.4668
Total number of RTI episodes	4.6 ± 0.3	4.3 ± 0.2	0.6037
Respiratory symptom duration (days)	7.8 ± 0.5	8.3 ± 0.5	0.9585
Weight Z-score at inclusion	− 1.4 ± 0.3	−1.4 ± 0.2	0.1166
Difference of weight Z-scores^a^	−0.5 ± 0.2	0.3 ± 0.1	0.5703
Cumulative number of days of antibiotics^b^	41.1 ± 5	37.8 ± 3.2	0.5646

Results are presented as mean ± SD. Wilcoxon signed rank for paired comparisons

^a^Weight Z-score at the end of the study period – weight Z-score at the beginning

^b^Over the year of the study follow-up

#### Viral RTI-related healthcare consumption

One hospitalization was directly related to a viral RTI (LRTI related to RV/EV–adenovirus co-infection) out of the five hospitalizations. The others causes of hospitalization were non-respiratory viral infection (gastroenteritis) in two cases, one for prolonged cardiorespiratory monitoring and one for surgery (hexadactylia).

Supplemental medications were needed in the vast majority of viral RTIs: oral antibiotics were prescribed at least once in 22 patients (88%) for a mean length of 13.8 ± 6.2 days and a mean cumulative number of days of 28.9 ± 21 days. Oral steroids were used in five patients (21%) for a mean length of 4.4 ± 1.4 days because of prolonged or recurrent wheezing spells, and bronchodilators were used in four (18%) for a mean length of 4.5 ± 7.8 days. A significant correlation was found between the total number of RTIs (virally proven or not) and the cumulative number of days under antibiotics used during the year of follow-up (Pearson correlation, *r* = 0.7941, *p* < 0.0001).

Viral RTI was associated with a mean 0 ± 0.2 days of parental absenteeism.

#### Nutritional impact

At the beginning of the study the mean weight Z-score was − 1.3 ± 1.4; it was − 0.9 ± 1.6 at the end of the study. There was no correlation between weight Z-score differences between the start and the end of the study and the number of viral RTIs occurring during the year (Pearson correlation, *r* = − 0.1708, *p* = 0.4142).

#### New pathogen acquisition

Four percent of the patients were positive for PA and 32% for SA at inclusion. A mean 5.6 ± 1.5 swabs were taken during the scheduled visit at the center for bacterial analysis. During the study, new isolation of PA occurred in five patients (5/24; 21%) and in ten patients (10/17; 59%) for SA. It is noteworthy that while none of our participants had *Stenotrophomonas maltophilia* (SM) at inclusion, four acquired this bacterium during the study period. The small number of patients in each subgroup precluded further statistical analysis. However, there was a trend for a higher number of total RTIs in patients with new isolations of PA, and the same was noticed for SM (Table 5).

**Table 5:** Microbiological outcome measures

	PA +	PA-	SA+	SA-	SM+	SM-
*n*	*5*	*20*	*10*	*15*	*4*	*21*
Total RTI number	4.2 ± 1.8	3.2 ± 1.7	3.3 ± 2.1	3.5 ± 1.6	4.8 ± 1.7	3.1 ± 1.7
Total virus positive RTI	1.0 ± 1.22	2.0 ± 1.6	1.1 ± 2.3	2.3 ± 1.5	2.5 ± 2.4	1.7 ± 1.4

PA+: patient with new isolation of *Pseudomonas aeruginosa* (PA) during the study period

PA-: patient without new isolation of *Pseudomonas aeruginosa* (PA) during the study period

SA+: patient with new isolation of *Staphylococcus aureus* (SA) during the study period

SA-: patient without new isolation of *Staphylococcus aureus* (SA) during the study period

SM+: patient with new isolation of *Stenotrophomonas maltophilia* (SM) during the study period

SM-: patient without new isolation of *Stenotrophomonas maltophilia* (SM) during the study period

## Discussion

### RTI epidemiology in young children with CF

Until recently, there were very few data describing RTI frequency, clinical consequences and nature of the virus recovered in young patients (≤ 2 years) with CF using molecular diagnostic tools [6]. A consortium has been set up between the USA and Australia to precisely evaluate the impact of early respiratory viral infections in young children with CF (NCT01973192). We found that a mean 3.4 RTI episodes occurred in our patients over 1 year; the maximum number of RTIs recorded was seven in one patient. This frequency is in accordance with data reported by Collinson et al. in the late 1990s (3.4 URTIs per year in children under 6 years of age) using cultural or serological tools [18], but is half the 3.8 episodes identified using PCR over the 6 winter months reported by van Ewijk et al. in a population of children with CF who were a mean 3.5 years old [9]. The most recent data published by the Swiss group did not provide a precise RTI frequency calculation because their methodology differed from ours [6]. The reason for the LRTI frequency reported in our study likely stems from the scoring system itself and the way we used it for the purposes of the study. This so-called “respiratory illness” scoring system was developed by van Ewijk et al. for parents for self-recording of URTI and/or LRTI clinical symptoms. This clinical score was systematically measured twice weekly. In the present study, because of the high risk of errors in filling in the diary or giving up over a longer period of time (12 months vs. 6 in the van Ewijk et al. study), we chose not to use it on a systematic basis, but rather as an aid for both parents and home caregivers to detect RTIs and launch nasal sampling. Furthermore, it appears that some RTI episodes may have been missed using this score, particularly mild URTIs.

As expected in this young population, RV/EV remain the most frequently recovered viruses during RTI. Since the spread of molecular diagnostic tools, many studies have reported the same results in older children [1, 4, 6, 9, 15, 25, 26], adolescents and adults [3, 10, 11, 15]. Overall, RV/EV are responsible for 70–80% of virally induced RTIs. However, we were more intrigued by the few RSV infections detected. Six patients were infected by RSV at a mean age of 14 ± 6.4 months; none required hospitalization, and even in the youngest patients, the clinical course was uncomplicated. These findings contrast with those reported decades ago in unscreened CF babies in whom RSV infection was described as severe (requiring oxygen supply or invasive ventilation) in three out of seven patients (43%) [16, 17, 27]. The overall better health status of the children with CF in the present sample is likely the main explanation for this result, as well as their older age at the time of RSV infection. These results are particularly interesting in terms of RSV infection prevention and subsequent clinical trials. Our population of patients was palivizumab-free. The American Academy of Pediatrics recommends the use of palivizumab in CF for targeted patients, particularly those with poor nutritional status or with several siblings [28]. We speculate that precautions and preventive measures (hand washing, family daycare attendance, avoidance of virally infected individuals, etc.) followed by parents (on medical advice and on their own initiative) may be efficient enough to reduce virus circulation around these patients.

### RV/EV carriage

Approximately one-third of the study population carried viruses without any symptoms. RV/EV were recovered in 51% of these cases. The clinical significance of this finding is currently unknown, as are the underlying mechanisms and potential consequences. Whether or not this was the same subtype of picornavirus (RV or EV) was not addressed in this study, but prolonged carriage has been reported [4, 29]. In a recent study conducted in a mean of 3.5 years old (range, 0–17 years) CF patients population sampled weekly for 6 months, the authors found that patients with CF had a more frequent and prolonged carriage of RV in comparison to their healthy counterparts, even if the subtypes did not differ [4]. The authors conclude that “this may indicate increased viral replication and/or decreased viral antiviral defense in patients with CF.” In younger patients (included within their first 3 months of life), Korten et al. found that RV without symptoms was detected in 20% of cases and that virus detection was less frequently associated with symptoms in comparison to healthy infants. This raises the question of the pathogenicity of these viruses in young patients with CF.

### Viral–bacterial interactions

We found that there was a trend toward a higher frequency of total RTI in young children in whom the first isolation of PA and SM occurred during the year of follow-up. However, we did not find the same trend for SA. However the study was not powered to specifically address this question, thus preventing us from drawing a firm conclusion. The risk factor that RTI represents for PA colonization was underlined several years ago, particularly after a severe episode of RTI [17, 27]. Furthermore, major pathogen acquisition in young children with CF (PA, SA*, Achromobacter xylosoxidans*) may follow seasonality, underpinning the concept of predisposing seasonal environmental factors such as respiratory virus epidemics [30, 31]. However, the trend identified herein for SM seems somewhat new and will need further attention. Whether viruses are able to directly alter airway bacterial flora in young patients with CF or these alterations are related to the frequent usage of antibiotics remains to be determined.

### Strengths and limitations of the study

This study’s strengths include its prospective cohort design, one of the first such studies conducted in young children with palivizumab-free CF, preventing potential interference of the monoclonal antibody on respiratory viral epidemiology. At home, samples were taken by physiotherapists using a detailed protocol, ensuring collection of good-quality samples.

This study also has limitations. (1) There were fewer samples in comparison to recent studies [4, 6]. (2) In our hands, despite the presence of healthcare providers at home, the scoring system used to help parents and professional caregivers identify RITs in children was weak and may have missed several mild RTIs. (3) The overall low number of samples in each subgroup make the analysis of some well-known risk factors of RTIs such as daycare attendance or tobacco smoke exposure impossible. (4) The subjects’ median age at inclusion was 6.4 months, which is likely already “too late” to evaluate the clinical impact of some viral RTIs that are known to be more worrisome at the youngest ages (usually before 3 months of age). (5) Finally, healthy controls were not included for comparison purposes.

## Conclusion and future work

This study shows that respiratory viruses are responsible for around two-thirds of RTIs in young children with CF. In contrast to older studies and in accordance with more recent studies, viral RTIs are usually of mild severity, exceptionally leading to hospitalization. RSV infections were rare, and this raises the question of the usefulness of RSV preventive medication in this young population.

## Data Availability

The datasets used and/or analysed during the current study are available from the corresponding author on reasonable request.

## Abbreviations

CF: Cystic fibrosis
EV: Enterovirus
LRTI: Lower RTI
PA: *Pseudomonas aeruginosa*
RSV: Respiratory syncytial virus
RTI: Respiratory tract infection
RV: Rhinovirus
SA: *Staphylococcus aureus*
URTI: Upper RTI
